# Application of high‐throughput sequencing for hereditary thrombocytopenia in southwestern China

**DOI:** 10.1002/jcla.23896

**Published:** 2021-07-08

**Authors:** Luying Zhang, Jie Yu, Ying Xian, Xianhao Wen, Xianmin Guan, Yuxia Guo, Mingzhu Luo, Ying Dou

**Affiliations:** ^1^ Department of Hematology and Oncology National Clinical Research Center for Child Health and Disorders Ministry of Education Key Laboratory of Child Development and Disorders Chongqing Key Laboratory of Pediatrics Children’s Hospital of Chongqing Medical University Chongqing China

**Keywords:** hereditary thrombocytopenia, high‐throughput sequencing, molecular diagnosis

## Abstract

**Background:**

The aim of this study was to design and analyze the applicability of a 21‐gene high‐throughput sequencing (HTS) panel in the molecular diagnosis of patients with hereditary thrombocytopenia (HT).

**Methods:**

A custom target enrichment library was designed to capture 21 genes known to be associated with HTs. Twenty‐four patients with an HT phenotype were studied using this technology.

**Results:**

One pathogenic variant on the *MYH9* gene and one likely pathogenic variant on the *ABCG8* gene previously known to cause HTs were identified. Additionally, 3 previously reported variants affecting *WAS*, *ADAMTS13*, and *GP1BA* were detected, and 9 novel variants affecting *FLNA*, *ITGB3*, *NBEAL2*, *MYH9*, *VWF*, and *ANKRD26* genes were identified. The 12 variants were classified to be of uncertain significance.

**Conclusion:**

Our results demonstrate that HTS is an accurate and reliable method of pre‐screening patients for variants in known HT‐causing genes. With the advantage of distinguishing HT from immune thrombocytopenia, HTS could play a key role in improving the clinical management of patients.

## Graphical abstract

A custom target enrichment library was designed to capture 21 genes known to be associated with HTs. Twenty‐four patients with an HT phenotype were studied using this technology. One pathogenic variant on the *MYH9* gene and one likely pathogenic variant on the *ABCG8* gene previously known to cause HTs were identified. Additionally, 3 previously reported variants affecting *WAS*, *ADAMTS13*, and *GP1BA* were detected, and 9 novel variants affecting *FLNA*, *ITGB3*, *NBEAL2*, *MYH9*, *VWF*, and *ANKRD26* genes were identified. The 12 variants were classified to be of uncertain significance.

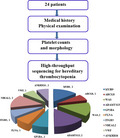

## 1 INTRODUCTION

Hereditary thrombocytopenias (HT) are a group of disorders characterized by spontaneous hemorrhage in the early postnatal period and excessive blood loss after trauma or surgery.^1^, ^2^ HTs represent thrombocytopenia and/or abnormal platelet function.^3^, ^4^ Due to the lack of specificity of clinical manifestations and screening methods, it is often misdiagnosed as immune thrombocytopenia (ITP).^5^, ^6^ Two important clinical characteristics for recognizing hereditary thrombocytopenia syndromes are the age of presentation and chronicity/duration of symptoms.^7^ In recent years, although significant progress has been made in the molecular pathogenesis of the disease, such as the discovery of abnormal gene expression in most patients, the pathophysiological mechanism of the disease is still unclear, and diagnosis is still difficult. In this study, we used high‐throughput target gene capture sequencing technology to establish a liquid‐phase capture chip of genes related to HTs in the form of a chip containing 21 genes known to be related to the disease. This microarray was used to detect these genes in children with a potential HT diagnosis. This study could provide a simple and feasible gene detection method for HT diagnosis in children in southwestern China, analyze the relationship between gene mutations and clinical characteristics, and provide a basis for the further study of pathophysiological mechanisms for HTs.

## 2 METHODS

### 2.1 Patients

Twenty‐four patients (15 males, 9 females; age range, 1 month to 13 years) from 24 unrelated southwestern Chinese families were enrolled in this study. All patients had a bleeding history. Most patients suffered from mild bleeding symptoms including cutaneous bruising, bleeding, and epistaxis, in addition to more severe bleeding symptoms in a few. Among the 24 patients in this study, 12 had been diagnosed with persistent or chronic ITP and had undergone ineffective treatments. The study was approved by the ethics committee of Children's Hospital of Chongqing Medical University, and informed consents were obtained.

### 2.2 Platelet counts and morphology

Platelet counts and morphology were studied in peripheral blood by sheath flow DC detection using the Sysmex XE‐2100.

### 2.3 DNA library preparation

For exome sequencing, we fragmented 1–3 μg of genomic DNA, extracted from each sample, to an average size of 180 bp with a Bioruptor Sonicator (Diagenode). Paired‐end sequencing libraries then were prepared using a DNA sample prep reagent set 1 (NEBNext). Library preparation included end repair, adapter ligation, and PCR enrichment and was carried out as recommended by Illumina protocols.

### 2.4 Targeted gene enrichment and sequencing

The amplified DNA was captured use GenCap Deafness capture kit (MyGenostics GenCap Enrichment technologies). The DNA probes were designed to tile along the exon regions of the thrombocytopenia genes. The original design included the following 21 genes: *MYH9*, *GP1BA*, *GP1BB*, *GP9*, *NBEAL2*, *vWF*, *GATA1*, *ABCG5*, *ABCG8*, *ITGA2B*, *ITGB3*, *FLNA*, *TUBB1*, *MPL*, *RBM8A*, *RUNX1*, *ANKRD26*, *HOXA11*, *CYCS*, *WIPF1*, and *WAS*. The capture experiment was conducted according to the manufacturer's protocol. In brief, 1 μg DNA library was mixed with Buffer BL and GenCap gene panel probe (MyGenostics, Beijing, China), heated at 95°C for 7 min and 65°C for 2 min on a PCR machine; 23 μl of the 65°C prewarmed Buffer HY (MyGenostics Inc., Beijing, China) was then added to the mix, and the mixture was held at 65°C with PCR lid heat on for 22 h for hybridization. 50 μl MyOne beads (Life Technology) was washed in 500 μl 1X binding buffer for 3 times and resuspended in 80 μl 1X binding buffer. Sixty‐four microlitres 2X binding buffer was added to the hybrid mix and transferred to the tube with 80 μl MyOne beads. The mix was rotated for 1 h on a rotator. The beads were then washed with WB1 buffer at room temperature for 15 min once and WB3 buffer at 65°C for 15 min three times. The bound DNA was then eluted with Buffer Elute. The eluted DNA was finally amplified for 15 cycles using the following program: 98°C for 30 s (1 cycle); 98°C for 25 s, 65°C for 30 s, 72°C for 30 s (15 cycles); 72°C for 5 min (1 cycle). The PCR product was purified using SPRI beads (Beckman Coulter) according to the manufacturer's protocol. The enrichment libraries were sequenced on Illumina HiSeq X ten sequencer for paired read 150 bp.^8^, ^9^, ^10^, ^11^, ^12^

### 2.5 Bioinformatics analysis

After sequencing, the raw data were saved as a FASTQ format and then followed the bioinformatics analysis: First, Illumina sequencing adapters and low‐quality reads (<80 bp) were filtered by cutadapt. After quality control, the clean reads were mapped to the UCSC hg19 human reference genome using BWA. Duplicated reads were removed using picard tools, and mapping reads were used for variation detection. Second, the variants of SNP and InDel were detected by GATK HaplotypeCaller, then using GATK VariantFiltration to filter variant, the filtered standard as follows: (a) variants with mapping qualities <30; (b) the total mapping quality zero reads <4; (c) approximate read depth <5; (d) QUAL < 50.0; (e) phred‐scaled *p*‐value using Fisher's exact test to detect strand bias >10.0. After above two steps, the data would be transformed to VCF format; variants were further annotated by ANNOVAR and associated with multiple databases, such as 1000 genome, ESP6500, dbSNP, EXAC, Inhouse (MyGenostics), HGMD, and predicted by SIFT, PolyPhen‐2, MutationTaster, GERP++.

### 2.6 Variants selected

In this course, five steps using to select the potential pathogenic mutations in downstream analysis: (a) Mutation reads should be more than 5, mutation ration should be no less than 30%; (b) removing the mutation, the frequency of which showed more than 5% in 1000 g, ESP6500, and Inhouse database; (c) if the mutations existed in InNormal database (MyGenostics), then dropped; and (d) removing the synonymous. (e) After (a), (b), (c), if the mutations were synonymous and they were reported in HGMD, left them. When finished above jobs, the mutations which were left should be the pathogenic mutations.

### 2.7 Sanger sequencing

Sanger sequencing has been used to validate variants of seven patients identified by the high‐throughput sequencing. The primers used have been listed in Table 1.

**TABLE 1:** Primer sequences of Sanger sequencing

Patient	Gene(s)	Chromosomal location	Primer sequences of Sanger sequencing
3	*MYH9*	chr22‐36691115	F‐5′‐AGCCTGTCTGAAGTCTGATGT‐3′
			R‐5′‐GCCTCTCTTTGGTCAGGGAA‐3′
9	*FLNA*	chrX‐153589716	F‐5′‐TTTAGGGCAGGTCTGGAGAAG‐3′
			R‐5′‐AAGGCCTTTGTCACATCCAG‐3′
10	*ITGB3*	chr17‐45331277	F‐5′‐GAGGAGCAATAGTTTCCCACC‐3′
			R‐5′‐CCAAGTCCGCAACTTGACC‐3′
11	*NBEAL2*	chr3‐47030600	F‐5′‐CTGCAGGTGTCTCTGTTGTCC‐3′
			R‐5′‐TCCTTTCAGCTGTAGGTGTGG‐3′
	*NBEAL2*	chr3‐47041758	F‐5′‐CAGATGTCTTCCTGCCCTCAG‐3′
			R‐5′‐CCACACCTTTGGAGAGGCTAC‐3′
13	*MYH9*	chr22‐36678719	F‐5′‐AGGAGGAGGCATGTTCACAG‐3′
			R‐5′‐CTTCTTTCTGGTGGGAGCAG‐3′
	*VWF*	chr12‐6230478	F‐5′‐CAGTGACCTTTCCGCTCAGAC‐3′
			R‐5′‐CTACGAGGCCAGAGAGGTTTG‐3′
14	*ANKRD26*	chr10‐27324137	F‐5′‐CAATAGCATGAAACTGGTCTTGG‐3′
			R‐5′‐TGGACGGCTTAGTGTTCTGAC‐3′
	*ANKRD26*	chr10‐27382670	F‐5′‐CTCTTCCTGGCATTGTACAGC‐3′
			R‐5′‐TACAATTTGGGATTTGGTTGG‐3′
18	*VWF*	chr12‐6138674	F‐5′‐ACCTACGATCAGGGAGCAGA‐3′
			R‐5′‐GTCATGGATGCCTGGAGAGT‐3′

## 3 RESULTS

### 3.1 Phenotyping of patient cohort recruited to the study

All 24 unrelated patients included in this study had various bleeding symptoms; 20 had mild levels of bleeding, and the remaining 4 had more severe bleeding symptoms including hemorrhage of the digestive tract or urinary tract, and menorrhagia. Three patients had a moderately low platelet count (20 × 10^9^/L < PLT < 50 × 10^9^/L), and 21 had an extremely low platelet count (20 < 10^9^/L). Four patients presented with a family history of thrombocytopenia. Patient 3’s father, patient 4’s mother, patient 9, and patient 15’s grandmothers experienced a history of thrombocytopenia. Twelve patients were diagnosed with persistent or chronic ITP and had a poor response to steroids or IVIG. Eight of them were identified as having HT‐specific gene variants. Detailed clinical symptoms and hematological characteristics of the 24 patients are displayed in Tables 2 and 3. The treatments and follow‐up of 12 patients with genetic abnormalities are displayed in Table 4.

**TABLE 2:** Phenotypic symptoms of 24 patients of unknown etiology

Patient	Age	Sex	Platelet count (×10^9^/L)	Platelet size	Bleeding phenotype	Associated findings	Diagnosed with persistent or chronic ITP	Family history of thrombocytopenia
1	9 months	M	6	NA	Cutaneous bruising, petechiae	None	No	No
2	3 years 7 months	M	20	Normal	Cutaneous bruising, petechiae	Hemolytic anemia	No	No
3	7 years	F	11	NA	Cutaneous bruising, petechiae, epistaxis	None	No	Yes
4	1 year 8 months	M	6	Normal	Cutaneous bruising, petechiae	None	Yes	Yes
5	5 years 4 months	M	18	NA	Cutaneous bruising, petechiae	None	No	No
6	2 months	M	17	Normal	Cutaneous bruising, petechiae	Hemolytic anemia, eczema, splenomegaly	No	No
7	1 year 3 months	F	17	Normal	Cutaneous bruising, petechiae	None	Yes	No
8	5 months	M	21	Normal	Petechiae	Diarrhea	Yes	No
9	1 year	M	49	NA	Hemorrhage of digestive tract	Recurrent infection, eczema, talipes equinovarus, hiatal hernia	No	Yes
10	9 months	M	10	NA	Cutaneous bruising, petechiae	None	Yes	No
11	4 years	M	12	Normal, slightly reduced	Cutaneous bruising, petechiae	None	Yes	No
12	10 months	F	4	NA	Cutaneous bruising, petechiae	Cytomegalovirus infection	No	No
13	1 year	M	11	Normal	Cutaneous bruising, petechiae	None	Yes	No
14	8 years	M	5	Normal, slightly increased	Cutaneous bruising, petechiae	None	Yes	No
15	8 months	F	4	Normal	Cutaneous bruising, petechiae	None	Yes	Yes
16	1 year	M	15	NA	Cutaneous bruising, petechiae	None	No	No
17	5 months	F	5	Normal	Petechiae	None	No	No
18	2 years	M	3	Normal, slightly increased	Cutaneous bruising, petechiae, epistaxis	None	No	No
19	1 month	M	23	NA	Petechiae	Eczema	No	No
20	3 months	F	6	Normal	Petechiae	None	No	No
21	13 years	F	4	Giant, large	Cutaneous bruising, petechiae, menorrhagia	None	Yes	No
22	4 years	F	8	Normal	Cutaneous bruising, petechiae	None	Yes	No
23	8 years	F	5	Normal	Cutaneous bruising, petechiae, epistaxis, urethrorrhagia	None	Yes	No
24	11 months	M	11	Normal	Cutaneous bruising, petechiae, hemorrhage of digestive tract	Eczema	Yes	No

Abbreviation: NA, not available.

**TABLE 3:** Hematological characteristics of 24 patients

Patient	WBC (10^9^/L)	HB (g/L)	Autoantibody	Coombs test	Immunoglobulin	Bone marrow cytology
1	5.8	118	Anti‐SSA antibody, anti‐Ro‐52 antibody, AMA M2: suspicious positive	Negative	Normal	The number of megakaryocytes increased with left shift of the nuclear
2	4.78	62	Negative	Negative	Normal	The number of megakaryocytes increased with the disorder of maturation, and erythroid proliferation was active, mainly in the intermediate and late erythroblasts
3	5.24	125	Negative	Negative	Normal	There were 103 megakaryocytes in the whole smear, 25 of which were classified and counted, including 7 juvenile megakaryocytes, 11 granular megakaryocytes, and 7 thromocytogenic megakaryocytes
4	4.43	109	Negative	Negative	Normal	Megakaryocytes increased without disorder of maturation
5	4.96	102	NA	Negative	NA	The number of megakaryocytes increased with the disorder of maturation
6	7.71	88	NA	NA	Normal	The number of megakaryocyte is not high, with the disorder of maturation, prolymphocyte accounted for 5.5%
7	4.11	117	Negative	Negative	Normal	The number of megakaryocytes increased (>300) and platelets could pile up
8	6.94	107	Negative	Negative	Normal	There were 579 megakaryocytes in the whole smear, no thromocytogenic megakaryocyte was found
9	6.66	73	Negative	NA	Low IgG level	The number of megakaryocytes increased with the disorder of maturation
10	5.47	110	Negative	NA	NA	The number of megakaryocytes increased with the disorder of maturation
11	4.73	130	Negative	Negative	Normal	The number of megakaryocytes was not significantly increased, but the maturation of megakaryocytes was impaired
12	5.23	84	Anti‐Ro‐52 antibody: suspicious positive	Negative	Normal	The number of megakaryocytes increased with the disorder of maturation
13	4.47	90	Negative	NA	Normal	There were 234 megakaryocytes in the whole smear, 25 of which were classified and counted, including 8 juvenile megakaryocytes and 17 granular megakaryocytes, megakaryocytes were not found, platelet is rare
14	12.03	82	Negative	Negative	Normal	The number of megakaryocytes increased with the disorder of maturation
15	1.9	78	Negative	Negative	Normal	The number of megakaryocytes increased with the disorder of maturation
16	4.65	102	Negative	NA	Normal	NA
17	5.2	113	ANA 1:100	Negative	NA	There were 1042 megakaryocytes in the whole smear, 25 of which were classified and counted, including 6 juvenile megakaryocytes, 17 granular megakaryocytes, and 2 thromocytogenic megakaryocytes, platelets scattered or small piles could be found
18	6.22	76	Negative	Negative	Normal	The number of megakaryocytes increased without disorder of maturation
19	13.2	74	Negative	Negative	Normal	The erythroid hyperplasia was obvious, prolymphocyte accounted for 4.5%
20	5.46	84	Anti‐SSA antibody, AMA M2: suspicious positive	Negative	Low IgA level	The number of megakaryocytes increased without disorder of maturation
21	11.03	103	Negative	Negative	Normal	The number of megakaryocytes increased with the disorder of maturation
22	10.02	117	Negative	Negative	Normal	The number of megakaryocytes increased with the disorder of maturation
23	5.18	68	Anti‐centromere antibody, AMA M2: suspicious positive	Negative	Normal	The number of megakaryocytes increased with the disorder of maturation
24	4.15	104	Anti‐SSA antibody, AMA M2: suspicious positive; anti‐Ro‐52 antibody: positive	Negative	NA	The number of megakaryocytes increased, thromocytogenic megakaryocytes decreased

Abbreviations: AMA, Anti‐mitochondrial antibody; ANA, Anti‐nuclear antibody; NA, not available.

**TABLE 4:** Treatments and follow‐up of 12 patients with genetic abnormalities

Patient	Treatments				Follow‐up
	Steroids	IVIG	Blood transfusion	Others	
3	No	No	No	No	PLT maintained at about 20 × 10^9^/L, no obvious bleeding, no organ function damage
9	No	Yes	RBC	Antibiotic	Died of gastrointestinal bleeding
10	Yes	Yes	No	No	Refused the follow‐up
11	Yes	Yes	No	No	Mucocutaneous hemorrhage, spleen slightly enlarged
12	Yes	Yes	No	No	PLT maintained at normal levels
13	Yes	Yes	No	No	PLT maintained at 20–30 × 10^9^/L, Cutaneous bruising, petechiae, no organ function damage
14	Yes	Yes	RBC	No	The steroid has not been stopped, and the platelet is normal
18	Yes	Yes	RBC	No	The steroid has been used for about one year. PLT maintained at about 10×10^9^/L. There were cutaneous petechiae and occasional hematoma after exercise, which could be relieved by themselves
21	Yes	Yes	RBC Platelet	Splenectomy	PLT maintained at 2–6 × 10^9^/L. Bleeding was obvious when during the menstrual period. Occasionally there were cutaneous petechiae. In May 2018, splenectomy was performed, and the platelet level increased after the operation
22	Yes	Yes	No	Traditional Chinese medicine	PLT maintained at about 20 × 10^9^/L without obvious bleeding
23	Yes	Yes	RBC	Traditional Chinese medicine	PLT maintained close to normal level without bleeding
24	Yes	Yes	RBC	Traditional Chinese medicine	PLT maintained above 30 × 10^9^/L in general, less than 30 × 10^9^/L when having a cold, and occasionally cutaneous petechiae were found

Abbreviation: RBC, red blood cell.

### 3.2 Candidate variants and variant prevalence in 12 patients

**FIGURE 1:**
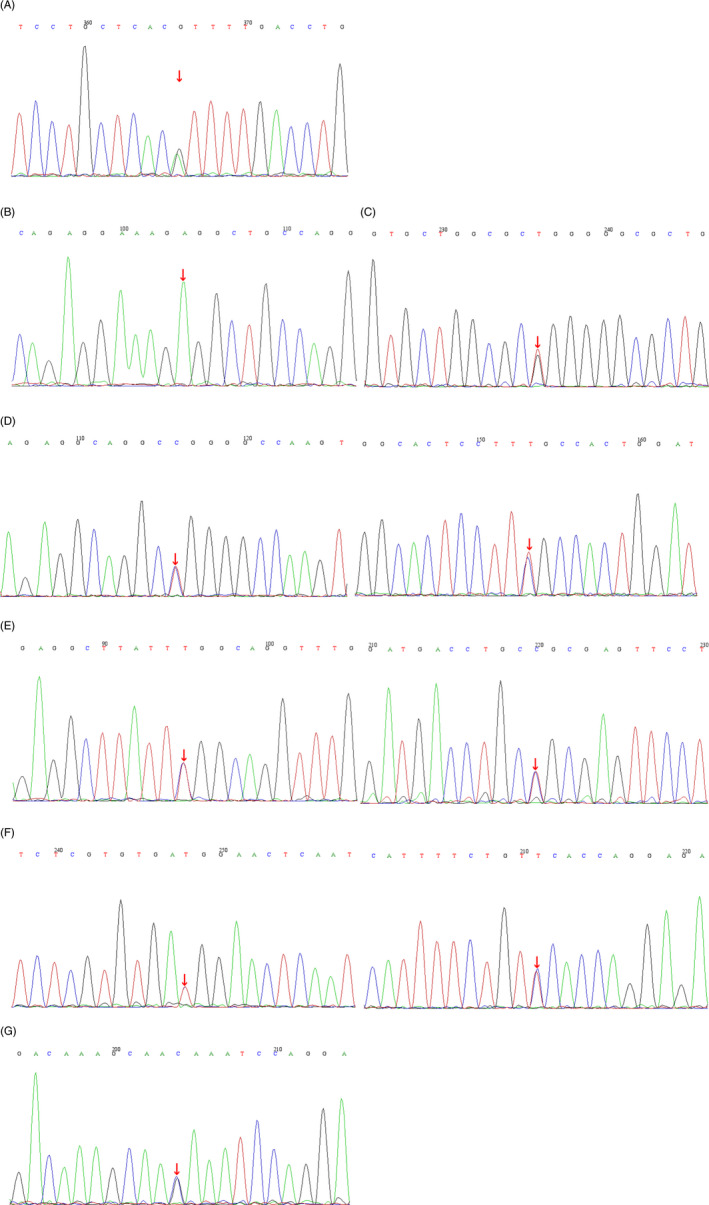
Sanger sequencing of the patients. (A) Patient 3: *MYH9* c.3493C>T, p.R1165C; (B) Patient 9: *FLNA* c.3167C>T, p.P1056L; (C) Patients 10: *ITGB3* c.50T>G, p.L17R; (D) Patient 11: *NBEAL2* c.4169C>T, p.S1390 L; *NBEAL2* c.295C>T, p.R99W; (E) Patient 13: *MYH9* c.5878G>A, p.E1960K; *VWF* c.82G>A, p.G28S; (F) Patient 14: *ANKRD26* c.3242A>G, p.H1081R; *ANKRD26* c.301G>A, p.D101 N; (G) Patient 18: *VWF* c.2823‐ 19G>C, splicing

In total, DNA samples from 24 patients were analyzed by a HT‐specific HTS panel. Following post‐sequencing bioinformatics analysis, candidate variants previously implicated in HT genes were observed in the patients. In total, 14 variants were noted in 12 patients, with a variant in a gene previously known to cause HT. One patient was observed with two variants in two different genes, and two patients were noted with two variants occurring within the same gene. Two variants were observed in a hemizygous state, and the others were observed in a heterozygous state. Thirteen of the variants identified were missense variants affecting a single amino acid. In addition, one splicing variant was noted in patient 18 (*VWF*; c.2823‐ 19G>C) (displayed in Table 5). Of the 14 variants, 10 were novel. Two known pathogenic or likely pathogenic variants were identified. These were found in patients 3 (*MYH9*; c.3493C>T, p.Arg1165Cys) and 23 (*ABCG8*; c.1877G>T; p.Gly626Val). Sanger sequencing confirmed *MYH9*, *FLNA*, *ITGB3*, *NBEAL2*, *VWF*, and *ANKRD26* variants among seven patients (displayed in Figure 1).

**TABLE 5:** Variants identified by analysis of the HT‐specific high‐throughput sequencing panel

Patient	Gene(s)	Transcript	Genomic variation	Protein effect	Variation type	Status	Inheritance	Classification	Allele frequency	Parents validation
3	*MYH9*	NM_002473	c.3493C>T	p.Arg1165Cys	Missense	Het	AD	Pathogenic	‐	NA
9	*FLNA*	NM_001110556	c.3167C>T	p.Pro1056Leu	Missense	Hemi	XR/XD	Uncertain significance	‐	Mother Het
10	*ITGB3*	NM_000212	c.50T>G	p.Leu17Arg	Missense	Het	AD	Uncertain significance	‐	Father Het
11	*NBEAL2*	NM_015175	c.295C>T	p.Arg99Trp	Missense	Het	AR	Uncertain significance	‐	Father Het
	*NBEAL2*	NM_015175	c.4169C>T	p.Ser1390Leu	Missense	Het	AR	Uncertain significance	0.0002	Mother Het
12	*WAS*	NM_000377	c.1378C>T	p.Pro460Ser	Missense	Het	XR	Uncertain significance	0.0058	NA
13	*MYH9*	NM_002473	c.5878G>A	p.Glu1960Lys	Missense	Het	AD	Uncertain significance	‐	Father Het
	*VWF*	NM_000552	c.82G>A	p.Gly28Ser	Missense	Het	AD/AR	Uncertain significance	‐	Mother Het
14	*ANKRD26*	NM_014915	c.3242A>G	p.His1081Arg	Missense	Het	AD	Uncertain significance	‐	Father Het
	*ANKRD26*	NM_014915	c.301G>A	p.Asp101Asn	Missense	Het	AD	Uncertain significance	0.0002	Mother Het
18	*VWF*	NM_000552	c.2823‐19G>C	splicing	splicing	Het	AD/AR	Uncertain significance	‐	Mother Het
21	*ADAMTS13*	NM_139025	c.2708C>T	p.Ser903Leu	Missense	Het	AR	Uncertain significance	0.0074	NA
22	*GP1BA*	NM_000173	c.1761A>C	p.Gln587His	Missense	Het	AD/AR	Uncertain significance	0.001	NA
23	*ABCG8*	NM_022437	c.1877G>T	p.Gly626Val	Missense	Het	AD/AR	Likely pathogenic	‐	NA
24	*WAS*	NM_000377	c.1378C>T	p.Pro460Ser	Missense	Hemi	XR	Uncertain significance	0.0058	NA

Abbreviations: AD, autosomal dominant; AR, autosomal recessive inheritance.

### 3.3 Pathogenicity prediction and variant classification

Of the 14 variants noted across all patients investigated, one variant was classified as “pathogenic” and one as “likely pathogenic” when considering the ACMG consensus guidelines. The remaining 12 variants, without a positive prediction of pathogenicity, were classified as of “uncertain significance.” A pathogenic variant was identified in 1 of 12 of patients, a likely pathogenic variant was identified in 1 of 12 of patients, and variants of uncertain significance were identified in 10 of 12 of patients.

## 4 DISCUSSION

Here, we present the first application of thrombocytopenia‐specific panel sequencing to patients with thrombocytopenia of unknown etiology in southwestern China. Platelet counts and phenotypic presentations varied considerably among the patients studied, which is consistent with the variability observed in the spectrum of HT.

The clinical features of the majority of patients with HT are mild to extremely low platelet count and various bleeding manifestations. Due to the lack of specific clinical symptoms other than low platelet count and a lack of convenient diagnostic methods, patients with HT are often misdiagnosed with ITP and receive unnecessary immunosuppressive therapy that could be ineffective. Among the 12 patients carrying gene variants discovered in this study, 8 had been misdiagnosed with ITP.

Among the 12 patients with HT variants found through HTS analysis, there were 14 gene variants detected, 10 of which were newly described. The pathogenic gene variant was identified in patient 3, which was a heterozygous missense variant c.3493C>T (p.Arg1165Cys) on the *MYH9* gene. *MYH9*‐RD is a type of HT disease caused by *MYH9* gene mutations. It is also a type of macrothrombocytopenia with a higher incidence than other types.^13^ *MYH9*‐RD variants were detected in 2 of the 12 patients in our study. Patient 3 with the reported variant c.3493C>T (p.Arg1165Cys) on the *MYH9* gene was a 7.1‐year‐old girl with mild bleeding symptoms since early life. The patient with the novel variant c.5878G>A (p.Glu1960Lys; case 13) was a one‐year‐old boy with repeated cutaneous bruising and petechiae. Both patients 3 and 13 had no kidney failure, hearing loss, or cataracts.^14^, ^15^

Case 9 had an unreported missense variant c.3167C>T (p.Pro1056Leu) on the *FLNA* gene. This patient was a 1‐year‐old boy who had reported hemorrhage of digestive tract and had developed talipes equinovarus and hiatal hernia. Localized variants in *FLNA* are believed to lead to a broad range of congenital malformations, affecting craniofacial structures, skeleton, brain, viscera, and urogenital tract.^16^ Hence, we consider that the variant we identified may be pathogenic.

Patient 10 was a rare case of a variant‐type GT in which the pathogenic variant was c.50T>G (p.Leu17Arg) on *ITGB3*. This variant was newly discovered and derived from the patient's father. According to Nurden's report,^17^ the primary feature of variant GT is a subtle reduction of the number of GPIIB/IIIa receptor molecules on the surface of platelets; however, the platelet aggregation function is defective, and some patients have reductions in their platelet counts. This patient was a nine‐month‐old boy with mild bleeding symptoms and a severely low platelet count.

Patient 11 had two unreported missense variants c.295C>T (p.Arg99Trp) and c.4169C>T (p.Ser1390Leu) on the *NBEAL2* gene, which related to Gray platelet syndrome.^18^, ^19^ This patient was a 4‐year‐old boy who had normal or slightly reduced platelet size rather than large platelet size. However, myelofibrosis or splenomegaly was not present. Patient 21 was a 13‐year‐old girl with mild bleeding symptoms since early life and manifested as menorrhagia when she came to our hospital. We found that she had a known risk variant in the *ADAMTS13* gene.^20^ However, she did not present with hemolytic anemia or nervous system symptoms, and we noticed that her platelet size was giant or large. Case 23 had gene variants in *ABCG8*. The missense variant c.1877G>T (p.Gly626Val) was previously reported^21^ and was classified as “likely pathogenic”. However, she did not have the manifestation of sitosterolemia. Patients 12 and 24 had the same previously reported risk variant in the *WAS* gene.^22^, ^23^ Because patient 12 was a ten‐month‐old girl, this variant in the *WAS* gene could not explain the disease. Patient 24 was an eleven‐month‐old boy who presented an X‐linked thrombocytopenia (XLT) phenotype including mild eczema. He did not experience recurrent infections. In addition, he showed detectable WASP expression.

Two unreported *ANKRD26* gene variants were identified in patient 14. *ANKRD26* expression is downregulated during megakaryocyte (MK) maturation by binding of RUNX1 and FLI1 to the 59 UTR of the gene. Pathogenetic mutations abolish this binding, resulting in *ANKRD26* overexpression in MKs, which, in turn, induces unbalanced activation of kinases downstream the MPL receptor, especially the MAPK/ERK 1/2 pathway. This mechanism induces altered MK maturation and reduced proplatelet extension.^24^ Thrombocytopenias caused by *ANKRD26* are characterized by predisposition to hematological malignancies.^25^ However, patient 14 did not develop a hematological malignancy.

A *GP1BA* variant c.1761A>C (p.Gln587His) was found in patient 22. At present, it is believed that the pathogenesis of Bernard‐Soulier syndrome (BSS) is due to a defect in biosynthesis and expression of platelet glycoprotein complex GPIb‐IX V or the defect of *GP1BA*, *GP1BB*, and *GP9* genes, which are important components of the complex. This results in platelets not adhering to the damaged vascular wall and a weakened response to thrombin, which lead to a variety of bleeding tendencies.^26^, ^27^ BSS tends to bleed obviously in the immediate postnatal period or childhood. It can worsen in adolescence or adulthood. It is characterized by decrease in platelet number and giant volume, the decrease in platelet aggregation induced by ristomycin,^28^ and normal platelet aggregation induced by collagen and ADP. However, patient 22 showed mild bleeding manifestation and normal platelet size when diagnosed.

There was a possible lack of genotype‐phenotype correlation shown in patients harboring mutations in *GP1BA*, *MYH9*, *ANKRD26*, and *ADAMTS13*. The patients represent a unique subset of each individual disease that does not share the typical phenotypic presentation of cases reported. However, further work would be needed to validate this.

Twelve patients in total were observed without any risk variants captured by the HT‐specific panel. Due to the absence of risk variants within the panel of 21 genes, there is a high chance that the genetic etiology of disease is due to variants in novel genes not previously implicated in HTs. Analysis of these patients, in particular, may progress our current knowledge of HTs through the determination of novel causative genes.^29^ Whole‐exome sequencing (WES) or whole‐genome sequencing (WGS) should be conducted for these patients in future studies. Further work should focus on platelet function as well.

## 5 CONCLUSION

This study demonstrates that HTS is an accurate and reliable tool for the genetic characterization of HTs. Due to the wide use of HTS, more hereditary thrombocytopenia‐associated gene variants have been discovered. It could become an important complement to first‐line diagnosis methods. Furthermore, implementing HTS in routine tests would elicit a more accurate diagnosis in patients with suspected hereditary thrombocytopenia. Patients with HT for whom HTS fails to identify the underlying molecular pathology are candidates for examination using less restrictive molecular approaches such as WES or WGS.

## CONFLICT OF INTEREST

The authors report no conflict of interest associated with this study.

## Data Availability

The data that support the findings of this study are available from the corresponding author upon reasonable request.
